# Master runners dominate 24-h ultramarathons worldwide—a retrospective data analysis from 1998 to 2011

**DOI:** 10.1186/2046-7648-2-21

**Published:** 2013-07-01

**Authors:** Matthias Zingg, Christoph Alexander Rüst, Romuald Lepers, Thomas Rosemann, Beat Knechtle

**Affiliations:** 1 Institute of General Practice and for Health Services Research, University of Zurich, Zurich, Switzerland; 2 Gesundheitszentrum St. Gallen, Vadianstrasse 26, St. Gallen 9001, Switzerland; 3 INSERM U1093, Faculty of Sport Sciences, University of Burgundy, Dijon, France

**Keywords:** Age of peak performance, Running speed, Gender difference

## Abstract

**Background:**

The aims of the present study were to examine (a) participation and performance trends and (b) the age of peak running performance in master athletes competing in 24-h ultra-marathons held worldwide between 1998 and 2011.

**Methods:**

Changes in both running speed and the age of peak running speed in 24-h master ultra-marathoners (39,664 finishers, including 8,013 women and 31,651 men) were analyzed.

**Results:**

The number of 24-h ultra-marathoners increased for both women and men across years (*P* < 0.01). The age of the annual fastest woman decreased from 48 years in 1998 to 35 years in 2011. The age of peaking running speed remained unchanged across time at 42.5 ± 5.2 years for the annual fastest men (*P* > 0.05). The age of the annual top ten women decreased from 42.6 ± 5.9 years (1998) to 40.1 ± 7.0 years (2011) (*P* < 0.01). For the annual top ten men, the age of peak running speed remained unchanged at 42 ± 2 years (*P* > 0.05). Running speed remained unchanged over time at 11.4 ± 0.4 km h^-1^ for the annual fastest men and 10.0 ± 0.2 km/h for the annual fastest women, respectively (*P* > 0.05). For the annual ten fastest women, running speed increased over time by 3.2% from 9.3 ± 0.3 to 9.6 ± 0.3 km/h (*P* < 0.01). Running speed of the annual top ten men remained unchanged at 10.8 ± 0.3 km/h (*P* > 0.05). Women in age groups 25–29 (*r*^2^ = 0.61, *P* < 0.01), 30–34 (*r*^2^ = 0.48, *P* < 0.01), 35–39 (*r*^2^ = 0.42, *P* = 0.01), 40–44 (*r*^2^ = 0.46, *P* < 0.01), 55–59 (*r*^2^ = 0.41, *P* = 0.03), and 60–64 (*r*^2^ = 0.57, *P* < 0.01) improved running speed; while women in age groups 45–49 and 50–54 maintained running speed (*P* > 0.05). Men improved running speed in age groups 25–29 (*r*^2^ = 0.48, *P* = 0.02), 45–49 (*r*^2^ = 0.34, *P* = 0.03), 50–54 (*r*^2^ = 0.50, *P* < 0.01), 55–59 (*r*^2^ = 0.70, *P* < 0.01), and 60–64 (*r*^2^ = 0.44, *P* = 0.03); while runners in age groups 30–34, 35–39, and 40–44 maintained running speed (*P* > 0.05).

**Conclusions:**

Female and male age group runners improved running speed. Runners aged >40 years achieved the fastest running speeds. By definition, runners aged >35 are master runners. The definition of master runners aged >35 years needs to be questioned for ultra-marathoners competing in 24-h ultra-marathons.

## Background

In running, a master athlete is defined as an athlete typically older than 35 years of age and systematically training for, and competing in, organized forms of sport specifically designed for older adults [1]. Master runners participate in endurance events all around the world [2-5] and accomplish top results up to approximately 50 years of age [2,4,5]. Especially in ultra-endurance events, where a variety of anthropometric [1,6,7], psychological [8], and environmental factors such as heat [9,10] may affect running performance, master runners have the chance to reach top rankings [11,12] and compensate age-related deficits such as the decrease in cardiovascular capacity [13].

The popularity of endurance and ultra-endurance running events such as the ‘New York City Marathon’ [2] or the ‘Western States 100-Mile Endurance Run’ [14] increased over the past years. Even if the annual number of athletes worldwide is by far smaller in ultra-marathons [15] than in marathons [16], thousands of runners complete each year ultra-marathons such as 24-hour ultra-marathons [17]. The increase in the number of athletes in both marathons [2,18] and ultra-marathons [11,19] is mainly due to an increase in the number of master runners and women [2,11,18,19]. An age-related decline in endurance performance has been shown for different running distances such as the marathon distance [18] and other types of running competitions [20-22] such as the 10-km run. Running speed in elite master runners showed a curvilinear decrease from the age of approximately 35 years until the age of approximately 60–70 years with an exponential decrease thereafter [23-25]. Peak running speed appeared to be maintained until the age of approximately 35 years [26,27].

However, in addition to the age-related performance decline, the age of peak endurance performance might also be of higher interest for athletes. The age of peak running speed was investigated in different running distances and in types of competitions such as marathons [28,29] and ultra-marathons [11,19]. For both women and men, the age of peak marathon performance has been reported to be at around 30 years [28], while in ultra-marathon events there seemed to be an increase in the age of peak performance across years into higher ages. For example, Eichenberger et al. [11] found in a 78-km mountain ultra-marathon, the ‘Swiss Alpine Marathon’ in Switzerland, an increase in the age of the annual top ten finishers from 33 years in 1998 to 37 years in 2011. Knechtle et al. [19] reported that the fastest 100-km running times were performed by men aged between 30 and 49 years and women aged between 30 and 54 years. Hoffman and Wegelin [14] showed that the fastest running times in a 161-km ultra-marathon were achieved by athletes aged between 35 and 40 years. Running speed beyond the age of 55 years seemed to decrease in both genders in studies investigating peak running performance for races with determined distances [13,29], but not for races with a time limit such as a 24-h ultra-marathon. To the best of our knowledge, no study investigated the age of peak running performance in 24-h ultra-marathons.

There might be differences in both the age-related performance decline and the age of peak endurance performance between women and men due to gender differences in performance. For running, the gender differences have been investigated for elite [28,30,31] and recreational runners [11,12]. Medic et al. [32] reported gender differences in performance in different sports such as swimming and track or field running and found them to be quite constant. Lepers and Cattagni [2] showed a relative stability of gender differences in marathon running times across the different age groups for the last decade in the New York City Marathon from 1980–2009 of about 11%. Cheuvront et al. [33] reported a gender difference of 8%–14% for running distances from 1,500 m to 42 km whereas the gender difference in running speed was approximately 11% in other competitions such as marathons [28,34]. A few studies investigated the gender difference in ultra-marathons [11,19] since it was hypothesized that women could outrun men in ultra-marathon distances or in a variety of other types of competitions [35,36].

Most studies investigated both the age-related decline in performance and the age of peak performance for a single race or a race series held within a country [2,4,5,11,14,19]. However, no study investigated the association of age and performance for an ultra-marathon race series held worldwide. The aims of the present study were to examine, first, the worldwide participation and performance trends of 24-h ultra-marathoners from 1998 to 2011, and second, to determine the age of peak running performance of these ultra-endurance runners. We hypothesized, first, that the participation would increase and the performance of master runners would improve over the 14-year period and, second, the age of peak 24-h running performance would be at around 40 years for both women and men with an increase in the age of peak performance across years.

## Methods

The present study was approved by the Institutional Review Board of St. Gallen, Switzerland, with a waiver of the requirement for informed consent given that the study involved the analysis of publicly available data. Performance and age of all men and women who ever participated in a 24-h ultra-marathon held worldwide between 1998 and 2011 were analyzed. The data set for this study was obtained from the race website http://www.ultra-marathon.org[17]. This data base collects all race results in ultra-marathon races held worldwide. Data before 1998 were not complete and were therefore seemed not reliable for data analysis.

### Data analysis

In order to facilitate reading and increase the comparability with similar analysis of races from different distances, race distances (km) were transformed to running speed (km/h) prior to analysis. Running speed (km/h) was calculated using the equation (running speed in km/h = race distance achieved in km)/24 h. To get the results as exact as possible, converting and further calculations were performed and are corrected to ten decimal places. To analyze the performance achieved in age groups, all athletes were divided into five-year age groups up to the age of 94 years prior to the analysis, starting with age group 18–24 years. To calculate the performance ratio and the gender difference, the annual top ten (e.g., annual ten fastest running speeds) women and men were determined from each age group, where all age groups were considered, providing at least ten athletes in both genders in more than ten out of the 14 analyzed years. The age groups 18–24 years and all age groups above 60–64 years had to be excluded from these analyses due to an insufficient number of athletes. Additionally, in the age group 25–29 years the results of the years 1998, 1999, and 2001, in the age group 55–59 years the results of the years 1998 and 1999, and in the age group 60–64 years the results of the years 1998, 1999, and 2002 had to be excluded from data analysis due to an insufficient number of athletes. Afterwards, the performance ratio for both genders and the gender difference in performance were calculated for each age group and year and analyzed regarding its change over time. Performance ratio was calculated using the equation (running speed of the top ten athletes in an age group / running speed of the overall top ten athletes) × 100. The performance ratio expresses the performance of the top ten athletes of an age group as a percentage of the performance of the overall top ten athletes. In order to compare the top ten athletes of an age group with the overall top ten athletes, the annual ten fastest times of each gender were collected. In order to determine the percentage of master athletes in the annual top ten, the top ten ranks from each year and both genders were collected. For the 14 years, 14 × ten top ten ranks were gathered for both genders, i.e., 140 top ten ranks for each gender. The gender difference in performance was calculated using the equation (running speed in women – running speed in men) / (running speed in men) × 100, where the gender difference was calculated for every couple of equally ranked athletes (e.g., between female and male winners, between women and men second place, etc.) before calculating mean value and standard deviation of all the pairings. In order to facilitate reading, all gender differences were transformed to absolute values prior to analyses. To analyze the age-related decline in running performance, the overall top ten women and men of all age groups comprised between 18–24 years and 75–79 years were determined and analyzed in the age period where the fastest performance was achieved.

### Statistical analysis

In order to increase the reliability of the data analyses, each set of data was tested for normal distribution and for homogeneity of variances prior to statistical analyses. Normal distribution was tested using a D'Agostino and Pearson omnibus normality test, and homogeneity of variances was tested using a Levene's. To find significant changes of a variable across years, linear regression was used. To find significant differences between multiple groups, a one-way analysis of variance (ANOVA) with subsequent Tukey-Kramer post-hoc analysis was performed. To determine the interaction between age and gender on running speed, a two-way ANOVA (age groups × gender) was used. Statistical analyses were performed using IBM SPSS Statistics (Version 19, IBM SPSS, Chicago, IL, USA) and GraphPad Prism (Version 5, GraphPad Software, La Jolla, CA, USA). Significance was accepted at *P* < 0.05 (two-sided for *t* tests). Data in the text are given as mean ± standard deviation (SD).

## Results

Data were available from 43,551 ultra-marathoners, including 8,767 women and 34,784 men. A total of 751 women and 3,125 men had to be excluded from data analysis due to missing information about age and eight more men and three women due to missing information about performance. Finally, data from 39,664 ultra-marathoners, including 8,013 women (20.2%) and 31,651 men (79.8%), could be included into data analysis.

### Participation trends

**Figure 1:**
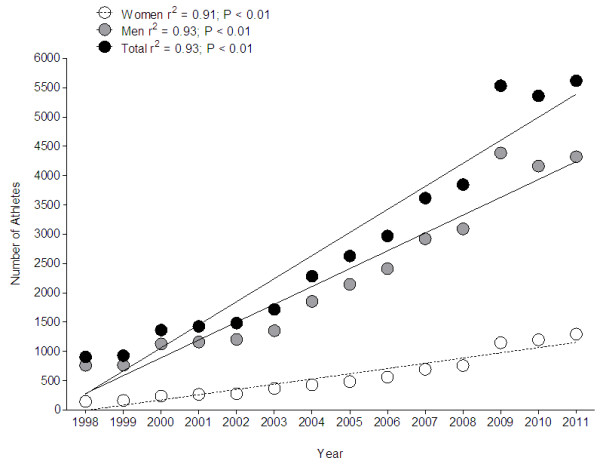
Changes in number of female and male athletes in 24-h ultra-marathons held worldwide from 1998 to 2011.

**Figure 2:**
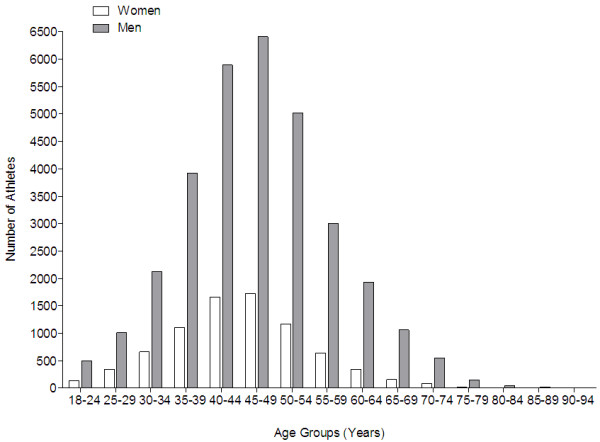
Number of women and men for different age groups in the 24-h ultra-marathons.

The numbers of finishers increased for both women and men over the 14-year period (Figure 1). The largest age group was athletes in the age group 45–49 years for both women and men (Figure 2). Among the 39,664 athletes, 86.0% were older than 35 years. Master runners aged >35 years achieved 120 (85.7%) of the 140 annuals top ten results in men and 129 (92.1%) of the 140 annuals top ten results in women. Master athletes from both genders achieved 27 out of the 28 fastest annual running speeds in 14 years. Women accounted on average for 20.2% of the field. The percentage of female participation increased from 16.0% in 1998 to 23.1% in 2011.

### Performance of the fastest 24-h runners

**Figure 3:**
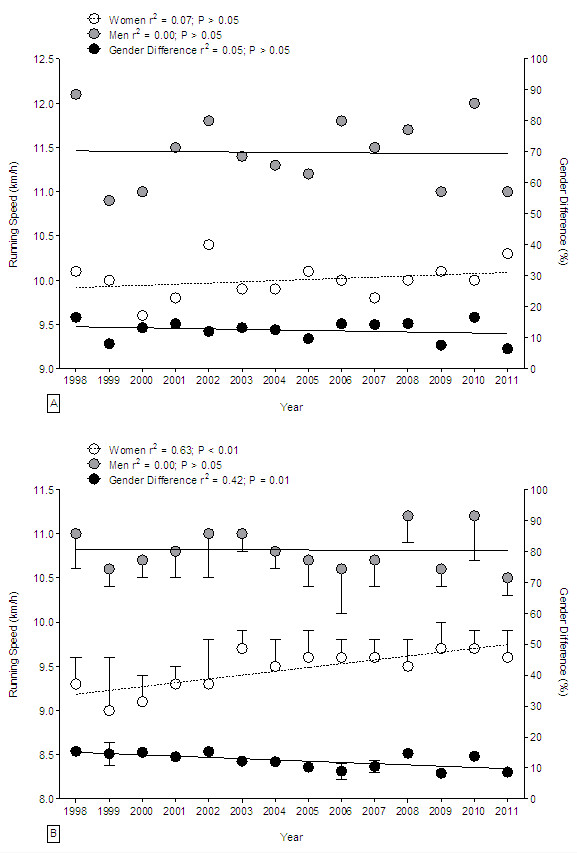
**Change in running speed in 24-hour ultra-marathons worldwide from 1998 to 2011. (A)** The annual fastest ultra-marathoners. **(B)** The annual ten fastest ultra-marathoners.

Running speed for the annual fastest 24-h ultra-marathoners remained unchanged across years in both women and men (Figure 3A). Mean running speed was 11.4 ± 0.4 km h^-1^ for the annual fastest men and 10.0 ± 0.2 km h^-1^ for the annual fastest women. The corresponding gender difference remained unchanged at 12.4% ± 3.3%. For the annual ten fastest female ultra-marathoners, running speed increased by 3.2% over the years (Figure 3B). In 1998 running speed for the annual top ten women was 9.3 ± 0.3 km h^-1^, and it increased to 9.6 ± 0.3 km h^-1^ in 2011. Running speed of the annual top ten men remained unchanged across years at 10.8 ± 0.3 km h^-1^ (*P* > 0.05). The difference in running speed between the annual top ten women and the annual top ten men decreased from 15.4% ± 0.5% (1998) to 8.7% ± 1% (2011).

### Age of the fastest 24-h runners

**Figure 4:**
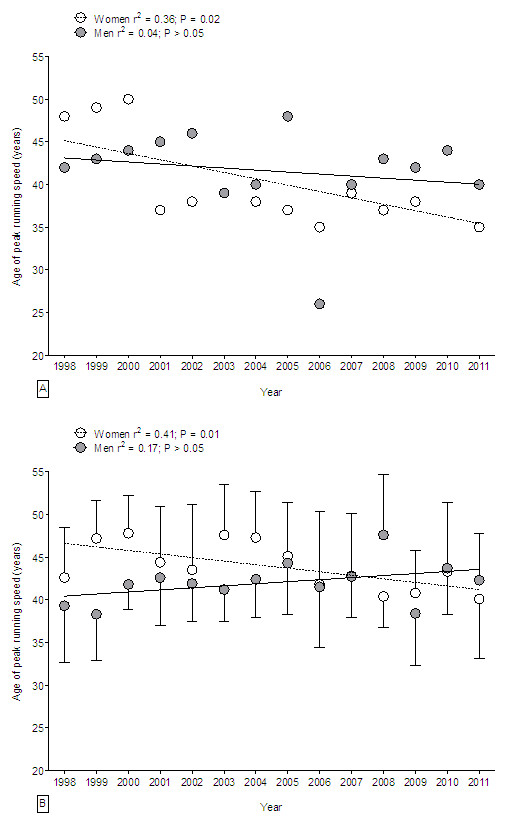
**Age of the fastest 24-h runners.** Change in the age of the annual fastest 24-hour ultra-marathoners **(A)** and the annual ten fastest 24-hour ultra-marathoners **(B)** from 1998 to 2011.

The age of peak running speed for the annual fastest 24-h ultra-marathoners was stable over the 14 years for men (*P* > 0.05), while it decreased for women (Figure 4A). The age of the annual fastest woman decreased from 48 years (1998) to 35 years (2011). The age of the annual fastest men (42.5 ± 5.2 years) showed no change over the 14 years (*P* > 0.05). For the annual top ten women and men, the age of peak running speed was unchanged for men (*P* > 0.05), while it decreased for women (Figure 4B). The age of the annual top ten women runners decreased from 42.6 ± 5.9 years (1998) to 40.1 ± 7.0 years (2011). The age of peak running speed of the annual top ten men remained unchanged at 42 ± 2 years (*P* > 0.05).

### Age-related change in performance

**Figure 5:**
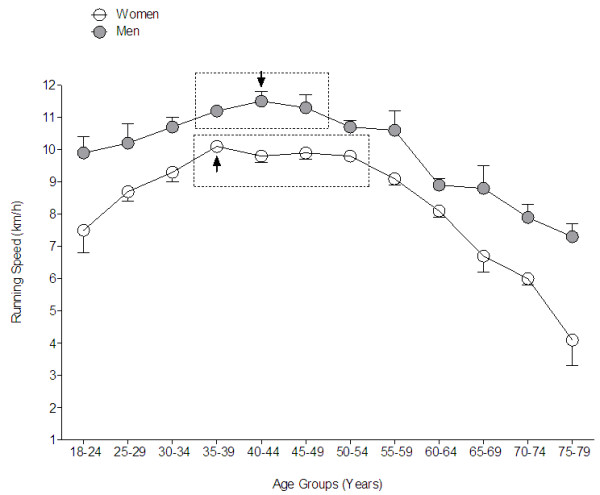
**Age-related change in 24-h ultra-marathon running speed of the overall top ten women and overall top ten men per age group (pooled data from 1998 to 2011).** An *arrow* indicates the age group with the fastest running speed. A *dotted line* indicates age groups which were not significantly different from the fastest one.

The age-related changes in performance over time are shown in Figure 5. Running speed decreased in a curvilinear manner with advancing age. There was a significant (*P* < 0.0001) age effect for both women (*F* = 234.4) and men (*F* = 103.8). No significant difference in running speed was observed for the three age groups between 35–39 and 45–49 years for men and the four age groups between 35–39 and 50–54 years for women (*P* > 0.05). In men, running speed was significantly (*P* < 0.0001) lower for the age groups 30–34 years and younger and 50–54 years and older compared with the age groups between 35–39 and 45–49 years. In women, running speed was significantly (*P* < 0.0001) lower for athletes in age groups 30–34 years and younger and 55–59 years and older compared with athletes in age groups between 35–39 and 50–54 years. Interaction analysis showed a significant age effect on running speed for both women and men (*F* = 13.64, *P* < 0.0001) where age accounted for 71.5% of the variance (*F* = 310.5, *P* < 0.0001) and gender for 20.8% (*F* = 992.5, *P* < 0.0001).

**Figure 6:**
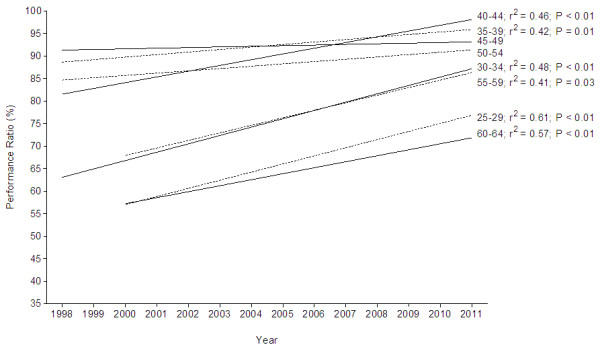
**Change in performance ratio for the different age groups for women from 1998 to 2011.** Performance ratio is expressed as percentage of the performance of the overall top ten athletes in the respective year. *r*^2^ and *P* values are inserted in case of a significant change in performance ratio over time.

Figure 6 shows the trends in performance ratio across years for the annual top ten runners subdivided into different age groups for women. Women in age groups 25–29 years (*r*^2^ = 0.61, *P* < 0.01), 30–34 years (*r*^2^ = 0.48, *P* < 0.01), 35–39 years (*r*^2^ = 0.42, *P* = 0.01), 40–44 years (*r*^2^ = 0.46, *P* < 0.01), 55–59 years (*r*^2^ = 0.41, *P* = 0.03) and 60–64 years (*r*^2^ = 0.57, *P* < 0.01) improved running speed over the 1998–2011 period, while women in age groups 45–49 years and 50–54 years maintained their running speed. The age group with the annual ten fastest women was the 40–44 years with an average of 92.7% ± 3.6% of the running speed of the overall top ten women. There was no decrease in the performance ratio.

**Figure 7:**
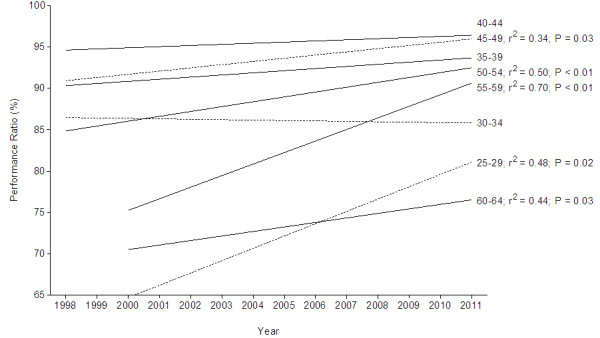
**Change in performance ratio for the different age groups for men from 1998 to 2011.** Performance ratio is expressed as percentage of the performance of the overall top ten athletes in the respective year. *r*^2^ and *P* values are inserted in case of a significant change in performance ratio over time.

Figure 7 presents the trends in performance ratio across years for the annual top ten male runners subdivided into different age groups. Men improved running speed in age groups 25–29 years (*r*^2^ = 0.48, *P* = 0.02), 45–49 years (*r*^2^ = 0.34, *P* = 0.03), 50–54 years (*r*^2^ = 0.50, *P* < 0.01), 55–59 years (*r*^2^ = 0.70, *P* < 0.01), and 60–64 years (*r*^2^ = 0.44, *P* = 0.03); while runners in age groups 30–34, 35–39, and 40–44 years maintained running speed. The fastest average running speed was accomplished by the top ten men in the age group 35–39 years with 96.1% ± 1.9% of the running speed of the overall top ten men. Also for men, there was no decrease in the performance ratio.

**Figure 8:**
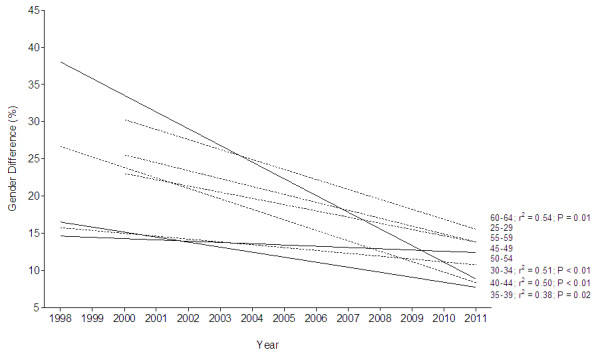
**Change in gender difference in 24-hour ultra-marathon running performance for each age group from 1998 to 2011.** R^2^ and *p* values are inserted in case of a significant change in gender difference over time.

Figure 8 presents the difference in time between the first and the tenth place for the annual top ten female and male runners. The gender difference in running speed decreased across years in age groups 30–44 and 60–64 years. The smallest gender difference in running speed was found in athletes aged 35–39 years with an average difference of 11.3% ± 4.6%, and the largest one in athletes aged 60–64 years with 23.2% ± 6.6%.

## Discussion

The aims of the study were to examine, first, participation and performance trends in 24-h ultra-marathons held worldwide between 1998 and 2011 and, second, the age of peak 24-h running performance. The main findings were, first, the number of finishers increased for both genders, second, the annual top ten women improved running speed, but not men, and, third, the age of peak running speed decreased for the annual top ten women, but not for men.

### Increase in participation in 24-h ultra-marathons

The number of finishers increased over years for both women and men. Running events are of high popularity with increasing numbers of participants [15-17]. The number of 24-h ultra-marathoners increased in both genders. Eichenberger et al. [11] reported similar findings for mountain ultra-marathoners competing in the 78-km Swiss Alpine Marathon held in Switzerland. The number of women increased between 2000 and 2011 but the number of men remained unchanged. Burfoot [3] reported an increase in the numbers of athletes in major marathons around the world such as New York City Marathon or ‘Boston Marathon’. These findings showed that master runners continue with an increased participation in both marathons and ultra-marathons worldwide.

Female participation was at 20.2% during this period and increased from 16.0% (1998) to 23.1% (2011). Similar findings were reported for 161-km ultra-marathoners competing in the Western States 100-Mile Endurance Run between 1974 and 2007 [14]. Women accounted for 10%–12% of the field in 1986–1988 and for 20%–22% of the field since 2001. Generally, men are over-represented in arts, sciences, and sports [37]. In distance running, more men than women run relatively fast [38] which is partly attributed to the greater training motivation in men [37]. Although female participation increased in US road races, the sex difference in performance remained stable [38]. A greater training motivation and a faster performance in men might be explained by the facts that most popular modern male sports require the skills needed for success in male-male physical competition, male champion athletes obtain a high status, and men pay closer attention than women to male sports [39].

### Runners over 35 years old achieved the best performances

Master runners aged >35 years achieved 120 (85.7%) of 140 annuals top ten results in men and 129 (92.1%) of 140 in women. Therefore, runners aged >35 years dominated 24-h ultra-marathons, while only a few runners younger than 35 years were able to keep up with the elite master athletes. The average age of the 140 top ten rankings was 43.4 years in women and 42.1 years in men, respectively. The age of peak running speed in a 24-h ultra-marathon was considerably higher than reported for marathons of around 30 years [28], of 33–37 years in 78-km mountain ultra-marathoners [11], and of 35–40 years for 161-km ultra-marathoners [14]. The definition of a ‘master athlete’ [1] needs to be revised in the near future since there seemed to be no difference in the running speed between the annual top athletes and master athletes.

### The gender difference in 24-h running speed

The gender difference in running speed in 24-h ultra-marathoners aged between 30 and 44 years decreased. Men maintained running speed in age groups between 30 and 44 years, whereas women increased their running speed in the age groups of 30–44 years across years. Younger athletes aged 25–29 years improved peak running speed in both genders which was quite unexpected. For example, Lepers and Cattagni [2] reported an improvement in running performance especially for master marathoners, but not for younger athletes. On the other site, older master athletes in the age group 55–64 years improved running speed in both genders, which is consistent with the findings of Lepers and Cattagni [2] for marathoners. Lepers and Cattagni [2] reported that running speed of male master marathoners within the 40–64 years age range have plateaued, while men older than 64 years and women older than 44 years improved running speed from 1980 to 2009. Since women increased and men only maintained running speed, the gender difference in performance decreased. Similar findings have been reported for marathoners. Lepers and Cattagni [2] showed that the gender differences in running times for marathoners decreased over the last three decades but remained relatively stable across the ages during the last decade.

Physiological differences between genders in maximal aerobic capacity (VO_2_max), skeletal muscle mass, velocity at lactate threshold, blood volume, maximum heart rate (HRmax), or stroke volume seemed to influence the gender difference [40-43]. For example, VO_2_max was found to be an important contributor to endurance performance in master runners [40], while VO_2_max was reported to differ among genders [41]. Since differences seemed to be quite constant between genders, VO_2_max seemed to be of biological origin [42]. Reaburn and Dascombe [1] reported VO_2_max to be the main reason for the decline in endurance performance; while other physiological variables such as HRmax, running velocity at lactate threshold, blood volume, or muscle mass seemed to be of lesser importance than VO_2_max.

A further important aspect of gender difference of running performance may lie in social aspects. The motivation in female runners differs from male runners [1,37-39,44], which could be an important reason for the differences in participation rates [2,37,38] and running performance [2,15-17]. Hodge et al. [8] found in master athletes enjoyment of their participation, a high commitment, high perceptions of ability and belonging, and an intrinsic motivation. These findings may explain the fact why master runners tend to participate in competitions not attracting much public attention and therefore require mostly intrinsic motivation.

### The age of peak running speed

Another important finding was that the age of the annual top ten men showed no change during the 14-year period whereas the annual top ten women became younger. Additionally, the age of the annual fastest men showed no change during the 14-year period, whereas the annual fastest women became younger. Generally, the fastest 24-h female ultra-marathoners became younger. Additionally, the beginning of the age range of peak running speed in life was equal for both women and men.

However, a difference can be found in the length of the time span where women and men could maintain their peak running speed. Women could maintain peak running speed longer in life up to 54 years. The top ten women could keep up peak running speed for a time span of 20 years (34–54 years), while the top ten male runners could maintain peak running speed only for 15 years (34–49 years). Knechtle et al. [19] found similar results for 100-km ultra-marathoners. They showed that female ultra-marathoners had a later decline in peak running speed at about 54 years of age than men at around 49 years. Tanaka and Seals [43] described peak endurance performance to be maintained until approximately 35 years of age, followed by a modest decrease until 50–60 years of age. Leyk et al. [4] similarly demonstrated that running speed showed no changes before the age of 50 years in marathoners and half-marathoners. An important limitation in investigating gender difference was the low number of female athletes especially in the earlier years.

The finding that the age of the annual top ten women decreased and the running speed of the annual top ten women improved is a new and unexpected finding. Different findings were reported for 161-km ultra-marathoners. Hoffman and Wegelin found in the Western States 100-Mile Endurance Run an increase in the age of the overall top five women and men between 1974 and 2007 where men showed no change in finish times but women have improved race times [14]. An improvement in female performance in the 24-h ultra-marathons might be explained with the increased female participation across the years. Some female finishers might have participated in several races for several years and gained experience and improved performance. Recent studies showed that previous experience such as personal best time was a strong predictor variable [45-48] in ultra-endurance performance.

A further important finding was the significant change in the gender difference in running speed across time. The annual fastest woman was on average about 13.2% slower than the fastest annual man, while the gender difference in running speed for the fastest annual athletes remained unchanged. The gender difference of the annual top ten athletes was 12.9% and therefore seemed to be in line with earlier reports. Cheuvront et al. [33] found the gender difference to be constant at 8%–14% in running races ranging from 1,500 to 42,195 m. In longer distances, Coast et al. [49] found over distances ranging from 100 m to 200 km a mean gender difference of 12.4%. Although it was speculated in the past that performances in both genders would eventually intersect and women could outrun men, these results show a difference in performance between genders. Krouse et al. [44] investigated motivation, goal orientation, and coaching in female ultra-marathoners. They reported that female ultra-marathoners were highly in task oriented and only little ego oriented. These intrinsic motivational factors may as well influence a relative constant gender difference in running speed.

### Why do master runners dominate ultra-marathon running?

The present findings showed that female and male master runners improved performance. Potential explanations for these findings could be motivational and social aspects [44,50], improvements in training [5], an increase in previous experience [1,45-48], and an increased longevity in the general population [51,52]. Knechtle et al. [53] reported that the personal best marathon running performance, but neither anthropometric nor training characteristics, were associated with performance in a 24-hour ultra-marathon. Therefore, all physiological parameters discussed earlier would not impact running performance in ultra-marathon distances as suggested by other authors [1,40-43]. Leyk et al. [54] found performance losses in middle age mainly to be due to a sedentary lifestyle, rather than biological aging. They showed that decline in performance was not related to age until the age of 55 years.

The aging society could provide another reason why the performance in master runners increased. For example, Robine and Paccaud [55] found an increase in the number of nonagenarians and centenarians in Switzerland as a result of a decreased mortality after the age of 80 years. But why do people get older in the general population? As the share of older people increased over the years [51,52], more master runners had the chance to participate in running competitions. Van Gool et al. [56] investigated between 1990 and 2007 in the Netherlands whether an increase in life expectancy was associated with a decrease in physical activity limitations. They reported that even though life expectancy increased, limitations of physical activity showed no decrease. Therefore, the gain of more years in life does influence the number of potential master runners but does not influence the level of their physical activity. Physical activities are known to differ between people living in a city or in the country [57,58]. Van Cauwenberg et al. [59] found in Belgium significant differences of habits of physical habits between rural and city citizens as they differed in type and quantity of physical exercise. Another important factor in aging and physical activity seemed to be nutrition [57]. Wagner et al. [57] showed that nutrition such as vitamin intake especially in seniors directly influenced their physical mobility. Healthy habits seemed to have an impact on life expectancy as earlier reported [59]. Among others, nutrition, non-smoking, and increased physical activity influenced mortality [59]. Master runners seemed not generally too old to accomplish astonishing feats in ultra-endurance running. Healthy habits such as continuous physical activities throughout life combined with training effort and commitment can compensate much of the age-related decrease in running performance.

### Limitations and implications for future research

This study is limited since variables such as physiological parameters [60], anthropometric characteristics [61,62], training data [63,64], previous experience [45-48,53,65] nutrition [66,67], fluid intake [68], environmental conditions [69-71], and motivational [37-39,44] factors were not considered. These variables may have had an influence on the race outcome. An athlete might have competed in several races a year and in the same race for several consecutive years. This might potentially have affected the analyses. Future studies need to investigate master runners in longer time-limited ultra-marathons such as 6 days and 10 days runs in case there would be enough runners aged 50 years or older.

## Conclusions

To summarize, the annuals fastest woman and the annual top ten women improved running speed over time while men only maintained running speed. The age of peak running speed of the annual fastest athletes was at approximately 38 years for women and at approximately 41 years for men. The annual top ten male athletes were approximately 41 years and the annual top ten female athletes 43 years of age. Therefore, the annual top runners were master runners by definition. The definition of master athletes >35 years needs to be questioned for ultra-marathoners. Future studies need to investigate longer time-limited competitions such as 48 h, 72 h, 6 days, and 10 days to investigate whether the age of peak running speed is even higher in longer races and increases with increasing length of the distance. The findings that the annual ten fastest women became faster and younger needs further investigation in other ultra-running distances.

## Abbreviations

HRmax: Maximum heart rate; VO2max: Maximal aerobic capacity.

## Competing interests

The authors report no conflicts of interest in this work.

## Authors’ contributions

MZ wrote the manuscript, CAR performed the statistical analyses, RL and TR helped in the statistical analyses, and BK collected the data. All authors read and approved the final manuscript.
